# On the internal reaction forces, energy absorption, and fracture in the hip during simulated sideways fall impact

**DOI:** 10.1371/journal.pone.0200952

**Published:** 2018-08-16

**Authors:** Ingmar Fleps, William S. Enns-Bray, Pierre Guy, Stephen J. Ferguson, Peter A. Cripton, Benedikt Helgason

**Affiliations:** 1 Institute for Biomechanics, ETH Zürich, Zürich, Switzerland; 2 Division of Orthopaedic Trauma, Department of Orthopaedics, University of British Columbia, Vancouver, Canada; 3 Orthopaedics and Injury Biomechanics Group, Department of Mechanical Engineering and Orthopaedics and School of Biomedical Engineering, University of British Columbia, Vancouver, Canada; 4 School of Science and Engineering, Reykjavik University, Reykjavik, Iceland; Universidad de Zaragoza, SPAIN

## Abstract

The majority of hip fractures have been reported to occur as a result of a fall with impact to the greater trochanter of the femur. Recently, we developed a novel cadaveric pendulum-based hip impact model and tested two cadaveric femur-pelvis constructs, embedded in a soft tissue surrogate. The outcome was a femoral neck fracture in a male specimen while a female specimen had no fracture. The aim of the present study was, first, to develop a methodology for constructing and assessing the accuracy of explicit Finite Element Models (FEMs) for simulation of sideways falls to the hip based on the experimental model. Second, to use the FEMs for quantifying the internal reaction forces and energy absorption in the hip during impact. Third, to assess the potential of the FEMs in terms of separating a femoral fracture endpoint from a non-fracture endpoint. Using a non-linear, strain rate dependent, and heterogeneous material mapping strategy for bone tissue in these models, we found the FEM-derived results to closely match the experimental test results in terms of impact forces and displacements of pelvic video markers up to the time of peak impact force with errors below 10%. We found the internal reaction forces in the femoral neck on the impact side to be approximately 35% lower than the impact force measured between soft tissue and ground for both specimens. In addition, we found the soft tissue to be the component that absorbed the largest part of the energy of the tissue types in the hip region. Finally, we found surface strain patterns derived from FEM results to match the fracture location and extent based on post testing x-rays of the specimens. This is the first study with quantitative data on the energy absorption in the pelvic region during a sideways fall.

## Introduction

Hip fractures pose a serious threat to the elderly, causing morbidity and an increased risk of mortality [1–3]. The majority of these fractures have been reported to occur as a result of a fall and impact on the greater trochanter (GT) of the femur [4]. The risk of sustaining a femoral fracture is thus affected by the probability of falling, the strength of the femur on the impacted side and the load applied to it in the event of a fall [5].

Subject specific finite element models (FEMs) of the proximal femur, loaded in a sideways fall configuration, can effectively predict femoral strength when compared to quasi-static (QS) ex vivo failure experiments on cadaver bones [6, 7]. However, QS loading is not an accurate reflection of the impact loads that cause the majority of hip fractures. A study comparing dynamic FEMs of the proximal femur that were subjected to an experimentally simulated fall, using a metal spring to model the compliance of the pelvis, found a high correlation between the measured and FEM-derived impulse response, and moderate correlations in femur stiffness and ultimate strength [8]. Although this demonstrates the ability of dynamic FEMs to simulate the effect an impact load has on the proximal femur, the question remains whether they can distinguish between a femur that will survive an impact resulting from a fall and a femur that will not.

Analytical models for predicting impact speed and fall alignment [9], combined with effective pelvic stiffness values [10], allow for an assessment of the impact loads generated as a result of a sideways fall from standing height, when combined with single degree of freedom (sDOF) spring-mass-dashpot models [11]. This approach is sensitive to pelvic stiffness, which has been measured on human volunteers using low-drop height experiments and found to increase non-linearly with increasing pelvis deflection [10]. This implies that using sDOF models with pelvic stiffness values derived solely from low energy falls could be a major limitation to accurately estimate higher impact loads. Depending on the model, input data, and subject specific parameters the predicted forces ranged from 1004–9990 N. [12] Furthermore, the sDOF models predict the impact reaction force at the boundary between the soft tissue and the ground, which is higher than the force transmitted through the impacted femur [13]. The only approach to overcome this limitation so far has been to estimate the internal force acting on the femur by using the impulse momentum principle. [14] The influence of soft tissue thickness on the measured force at the femoral neck was studied by Robinovitch et al. [15]. They used a pendulum experiment that impacted onto a cadaveric soft tissue model with surrogate hard tissue structures and found the force at the femoral neck to decrease with increasing soft tissue thickness, while keeping the impact energy, impact speed and structural stiffness constant.

To overcome the limitations associated with low drop height studies, dynamic subject-specific FEMs of male femur-pelvis-soft tissue constructs have been developed and used to quantify the influence of GT soft tissue thickness, impact speed, subject height and weight on the resulting impact force between the soft tissue and ground [16–18]. These studies provided an increased understanding of the mechanics of impact to the hip, despite some limitations associated with the FEMs, such as all joints being modelled as rigid. The modelling technique was partially validated by Majumder and Roychowdhury [16], who found a high correlation between measured and FEM predicted impact force but with the FEMs over predicting it by 29% on average. In these experiments, the specimen was resting sideways on the floor while a falling mass impacted the GT on the upper side with gradually increasing input energy. However, it is unclear how well this experimental setup represents real life sideways fall to the hip where the pelvis is moving and has considerable momentum in a dynamic system.

The energy absorption capability of a human femur has been reported to be roughly an order of magnitude lower than the total energy associated with an impact during a sideways fall from standing height [19]. For understanding what predisposes a particular hip to fracture, it is thus important to determine where energy is absorbed in the tissues of the numerous individuals that do not fracture their hip during a fall. Increasing the biofidelity of cadaveric experiments that focus on hip fracture, by applying impact energy to the specimens in such a manner that it accurately represents the fall event, is thus of considerable interest. Recently, we developed a novel cadaveric pendulum-based hip fracture model and tested two ex vivo cadaver femur-pelvis constructs with ligaments intact, embedded in a soft tissue surrogate made of ballistic gel [20]. The outcome of these tests was one femoral neck fracture and one non-fracture. This opens up the possibility of using FEMs of these tests to investigate aspects of the hip fracture and non-fractures that are difficult to study experimentally, and assess the potential of the FEMs in terms of predicting the outcome of falls.

The aim of the present study was therefore threefold. First, to develop a methodology for constructing and assessing the accuracy of FEMs for biofidelic simulation of sideways falls to the hip that is based on our previously reported experimental model. Second, to use, for the first time to our knowledge, explicit FEMs for quantifying the internal reaction forces and energy absorption in the hip during impact as a result of these falls. Third, to assess the potential of the FEMs in terms of separating a femoral fracture endpoint from a non-fracture endpoint.

## Materials and methods

### Experimental testing

**Fig 1:**
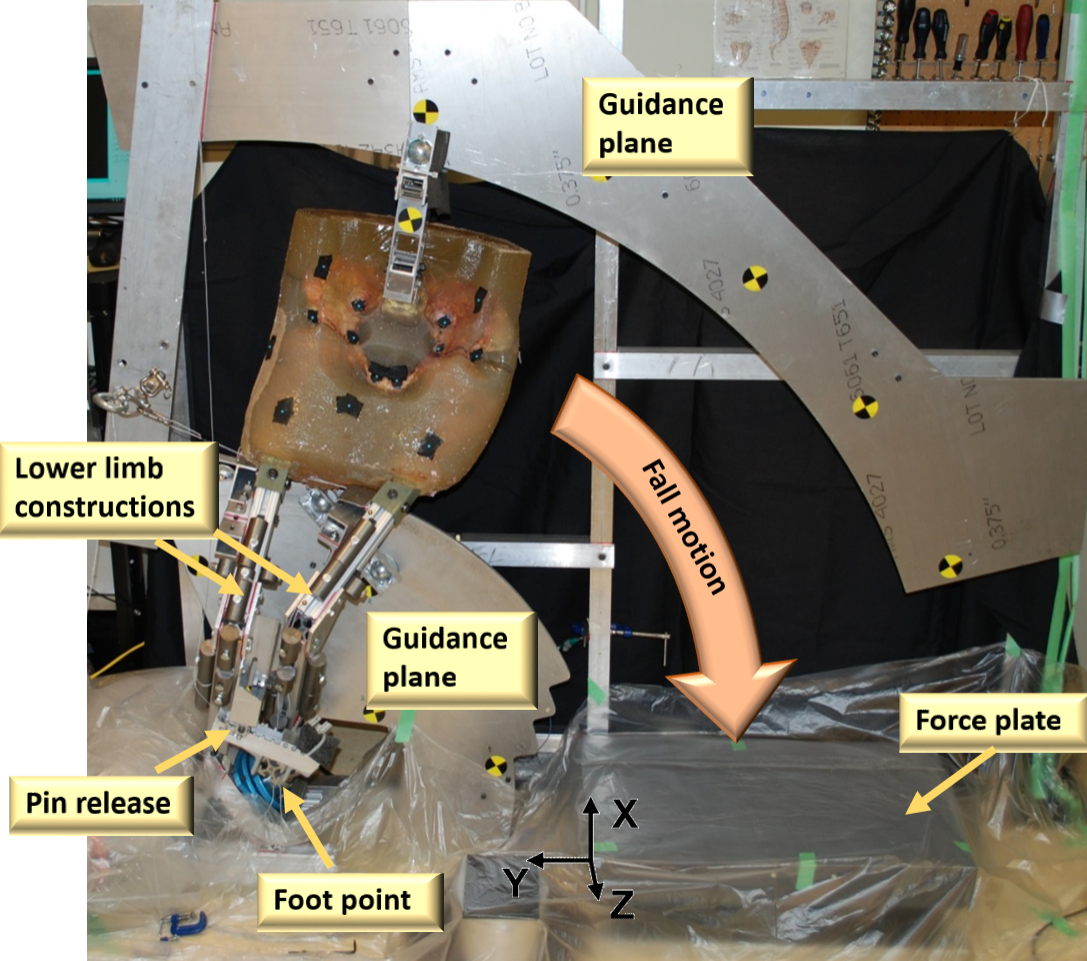
Experimental setup of the pendulum drop tests (reprinted from Fleps et al. [20]). Two planes were used to guide rollers attached to the lower limbs and pelvis. These guides constrained the specimen during the fall phase to a single degree of freedom. Cut guidance planes allowed for a free impact of the specimens, which were only constrained by the ball and socket joint at the foot point at impact. A pin-release constrained the motion of the contralateral leg during the fall phase but released it before impact. Rigid steel masses were added to the lower limb constructs to achieve subject specific target limb masses for each specimen. [22].

A detailed description of the pendulum fall simulator experiment can be found in Fleps et al. [20]. A brief description is provided here for clarity and context. Pelvic cadaveric specimens were embedded in surrogate soft tissue and attached to surrogate lower limb constructions that were designed to mimic the subject’s thighs and calves. The soft tissue shapes were generated by matching subjects in an existing body shape database (SizeUSA, [TC]^2^ Labs, Apex, NY, USA) and therefore allowed the test specimens to be BMI-matched to the donor. The specimens were subjected to a guided pendulum motion with a one degree-of-freedom rotational motion to simulate an unprotected fall to the side. All constraints imposed by the guides were released directly before impact except for three-dimensional translational constraints at the foot point of the impacted leg. This experimental setup is illustrated in Fig 1.

**Fig 2:**
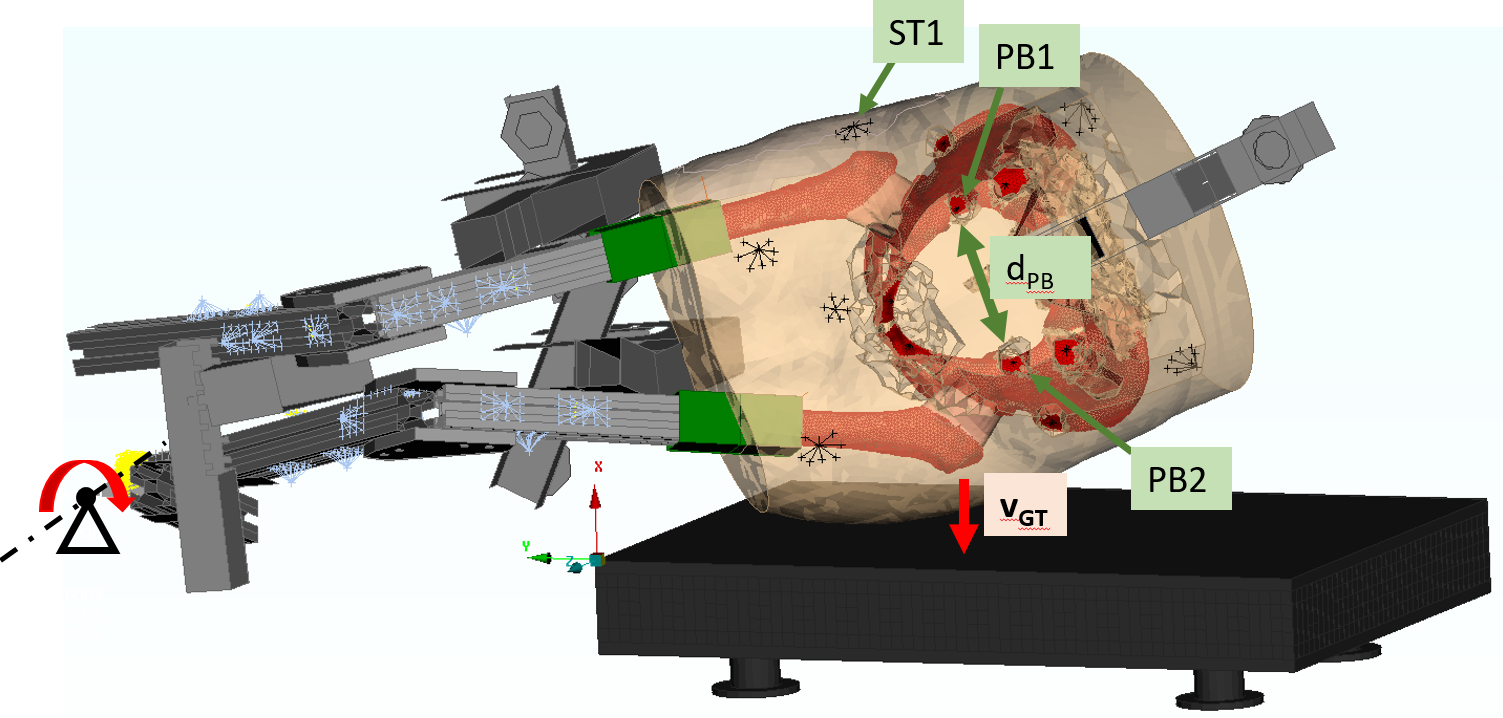
FEM of specimen H1391 aligned in initial position corresponding to the time of first contact with force plate during the experimental testing. Lower limb constructions coloured grey, lower limb masses coloured blue and soft tissue coloured orange. Marker points corresponding to location of video markers of the physical specimen are shown in black. Marker ST1 corresponds to a marker attached to the surrogate soft tissue above the right GT. Markers LR1 and LR2 correspond to the position of markers attached to the right and left side of the pelvic ring, respectively.

The study was approved by the University of British Columbia Clinical Research Ethics Board (Study ID H06-70337). Specimen preparation and experiments were conducted at the UBC Centre for Hip Health and Mobility and the International Collaboration On Repair Discoveries (ICORD) centre. Two specimens, one male and one female, were tested. Consent was given either written or electronically by the donor or next of kin. Detailed specimen information is provided in Table 1. (More detailed information is provided in the S1 Detailed specimen information) Alignment and mass locations were carefully documented for the specimens prior to testing. The impact force-time response during the fall simulation was recorded using a 6-axis force plate running at 10, 000 Hz (Bertec, Columbus, Ohio, USA). Two high-speed (HS) video cameras running at a frame rate of 5000 Hz (Phantom v12, Vision Research, Wayne, New Jersey, USA) were used to track 8 markers attached to the bones, with an emphasis on collecting deformation of the pelvic inlet, and 6 markers attached to the surrogate soft tissue. The displacement of all markers was tracked using the HS video data to quantify their position, which enables comparison to FEM-derived results. The markers labelled LR1 and LR2, attached to the left and right side of the pelvic ring respectively, and the marker labelled ST1, attached to the surrogate soft tissue above the right GT, were of particular interest (see Fig 2). Tracking marker ST1 allows for assessment of the effective pelvic stiffness of the specimens comparable to the pelvic stiffness derived from low drop height studies [10]. Tracking markers LR1 and LR2 allows for an assessment of the pelvic ring compression which has been reported in the past in impact tests on cadaveric pelvises [21]. Specimen alignment was measured prior to each test with a digitizing probe. The decent of the specimen was dynamically monitored during each test with two markers on the impacted leg, sampled at 900Hz (OptoTrack Certus system, Northern Digital Inc, Waterloo, Canada). This allowed for determination of the impact speed at the greater trochanter (V_GT_) of the specimens at the time of the initial contact with the force plate. Pre- and post-testing x-rays were acquired for fracture identification. The experiments resulted in a clinically relevant femoral neck fracture in the male specimen (H1406). No visible fracture could be found in the female specimen (H1391).

**Table 1:** Specimen information. **Donor data is shaded in green, experimental impact parameters are shaded in orange and experimental results are shaded in blue**. (a more detailed table can be found in S1 Detailed specimen information).

Parameters	Unit	H1391	H1406
Gender		Female	Male
Age	[years]	62	94
Height	[m]	1.65	1.73
Body mass	[kg]	59.0	83.9
Femoral neck aBMD	[g/cm^2^]	0.694	0.697
T-score	[]	-0.91	-0.88
Rotational impact velocity	[1/s]	4.13	4.15
Inertia I_Z_ with respect to foot point	[kg*m^2^]	12.9	19.6
Specimen mass, pendulum	[kg]	30.56	44.35
Rotational energy, pendulum	[J]	104.1	169.0
Greater trochanter soft tissue thickness	[mm]	32	31
Peak impact force	[N]	5641	7132
Fracture status		Non-fracture	Fracture

### Finite element models (FEMs)

Dynamic non-linear Finite Element models (FEMs) of the specimens were constructed and solved using commercial software (LS-Dyna, Livermore, USA). The models, which contained 785 238 elements (264691 Nodes) and 1 004 856 elements (299 871 Nodes) for specimen H1391 and H1406 respectively, were built using a commercial pre-processor (Ansa 17.1.0, Beta CAE Systems, Switzerland). They were solved on a computational cluster using 4 CPUs in parallel. Simulating 40 ms of the impact took around 30 hours of wall time per model.

#### FEM construction

Clinical resolution CT scans (Toshiba Aquilion One, Ōtawara, Tochigi, Japan) (120 kVp, 200 mAs, voxel size: 0.78 mm X 0.78 mm X 0.3 mm, slice thickness 0.5 mm) were acquired for both specimens prior to testing. A hydroxyapatite phantom (QC1, Scanco Medical) was scanned with each specimen to calibrate CT grey scale values to bone density. Simulated areal bone mineral density (aBMD) values for the femoral neck (Table 1) were calculated using commercial software (QCTPro, Texas, USA). The 3D geometry of markers, bones, and mechanical fixations were segmented from the CT image using an open source software (MITK-GEM, https://simtk.org/projects/mitk-gem) [23].

Cartilage geometry at the hip joints, pubic symphysis and sacroiliac joints was derived based on extracted bone geometry. The articular cartilage at the hip was modelled with a single element layer in the joint space, attached to the acetabulum, to avoid the use of small elements. A frictionless contact was modelled between the femoral head and the cartilage layer. This enabled realistic joint constraints dictated by cartilage, ligament and bone compliance. At the pubic symphysis and the sacroiliac joints the cartilage was bonded to the bone thus constraining the small translational motions that are physiologically possible at the sacroiliac joint. The femoral capsule and ligaments were modelled based on anatomical literature and photographs taken of the cadaveric specimens at the time of dissection.

Soft tissue geometry, position, and mass in the FEMs were kept in close agreement with the experimental setup with help of the digitizer data gathered from the experimental setup just prior to testing. The lower limb constructions, including subject-specific masses and guidance system configuration were modelled based on dimensions, masses, position and alignment measured prior to each impact test. The pelvic guidance roller was also modelled based on experimental masses and geometry. The force plate was modelled as a rigid structure.

#### FEM mesh parameters

Tetrahedral elements with an average edge length of 3 mms were used to model bone and cartilage. The femur on the impact side was modelled with 10 node tetrahedral elements, all other cadaveric tissues were modelled with 4 node tetrahedral elements. The lower limb constructions were modelled with first-order shell elements with a mesh density of 5 mm, except for the PMMA volumes, in which the distal end of the femurs were embedded, which were also modelled with 4 node tetrahedral elements. The bulk soft tissue was modelled with 4 node tetrahedral elements with a mesh gradient from 3 mm at the bone surface to 10 mm at the outer surface. Mesh convergence for the impacted femur was tested in a separate study [8]. The yield and fracture force of the femur was found to be mesh dependent, which dictates the element size used to model the bones in the present study. A refinement of the soft tissue mesh from 10 to 5 mm did not result in any significant changes in the system response. A mesh density with a gradient from 3 to 10 mm was chosen to reduce computational cost. The hip capsule ligaments were modelled with a combination of shell elements and tension only cable elements. The cable elements were applied to all shell edges, therefore spanning a mesh of tension only elements over the whole capsule. Pelvic ligaments were modelled with tension only cable elements, spanning between attachment sites. At the ligament attachment sites an asterix of beam elements was used to distribute the load over a wider surface area.

#### Material properties

The material mapping strategy was previously developed by Enns-Bray et al. [8]. It is briefly described here for clarity. Heterogeneous material properties were mapped to the elements of the bone volumes based on the calibrated CT grey scale values using the material mapping method “B” from Helgason et al. [24]. The modulus-density relationship *E* = 6850 ∙ *ρ_app_*^1.49^ from the study of Morgan et al. [25] was used, with 500 equidistant stiffness values in the range of 0.01–22 GPa. Non-linear material behaviour with tension compression asymmetry was implemented using a Fu-Chang Foam model (LS-Dyna, MAT_083). Strain rate dependency of the mechanical properties of cancellous bone was based on the findings of Carter and Hayes [26] and for cortical bone on the findings of Hansen et al. [27]. Element erosion was implemented for elements representing bone if volume strains exceeded +20%. Element erosion was only implemented for stabilizing the solution process at large deformations. No difference in predicted peak impact force was found for simulations with and without element erosion.

Acetabular cartilage was modelled based on the study of Burgin et al. [28] using a hyperelastic material model without viscoelastic effect (LS Dyna, MAT_077, v = 0.495, rho = 0.795 g/cm^3^, C10 = 0.352 MPa, C01 = 0.306 MPa, C11 = 0.052 MPa). Hyperelastic material parameters for fibrous cartilage at the pubic symphysis were based on the study from Li et al. [29] (LS Dyna, MAT_027, v = 0.495, rho = 0.795 g/cm^3^, C10 = 0.1 MPa, C01 = 0.45 MPa). The same parameters were also used for the cartilage in the sacroiliac joint for lack of better published information. A linear elastic material with modulus of elasticity of 0.002 MPa, was used to model the matrix of the capsule, which was modelled with shell elements. Cable elements were implemented with a toe region of 8% strain based on Hewitt et al. [30] and a linear fibre stiffness of 200 MPa (LS Dyna, MAT_071). This resulted in significant stiffness in tension while providing minimal resistance in compression. Pelvic ligaments were modelled with tension only cable elements with material properties according to Hammer et al. [31] (LS Dyna, MAT_071, E = 395 MPa). Cross sections that were based on subject specific insertion site length and an average ligament thickness of 1mm.

**Fig 3:**
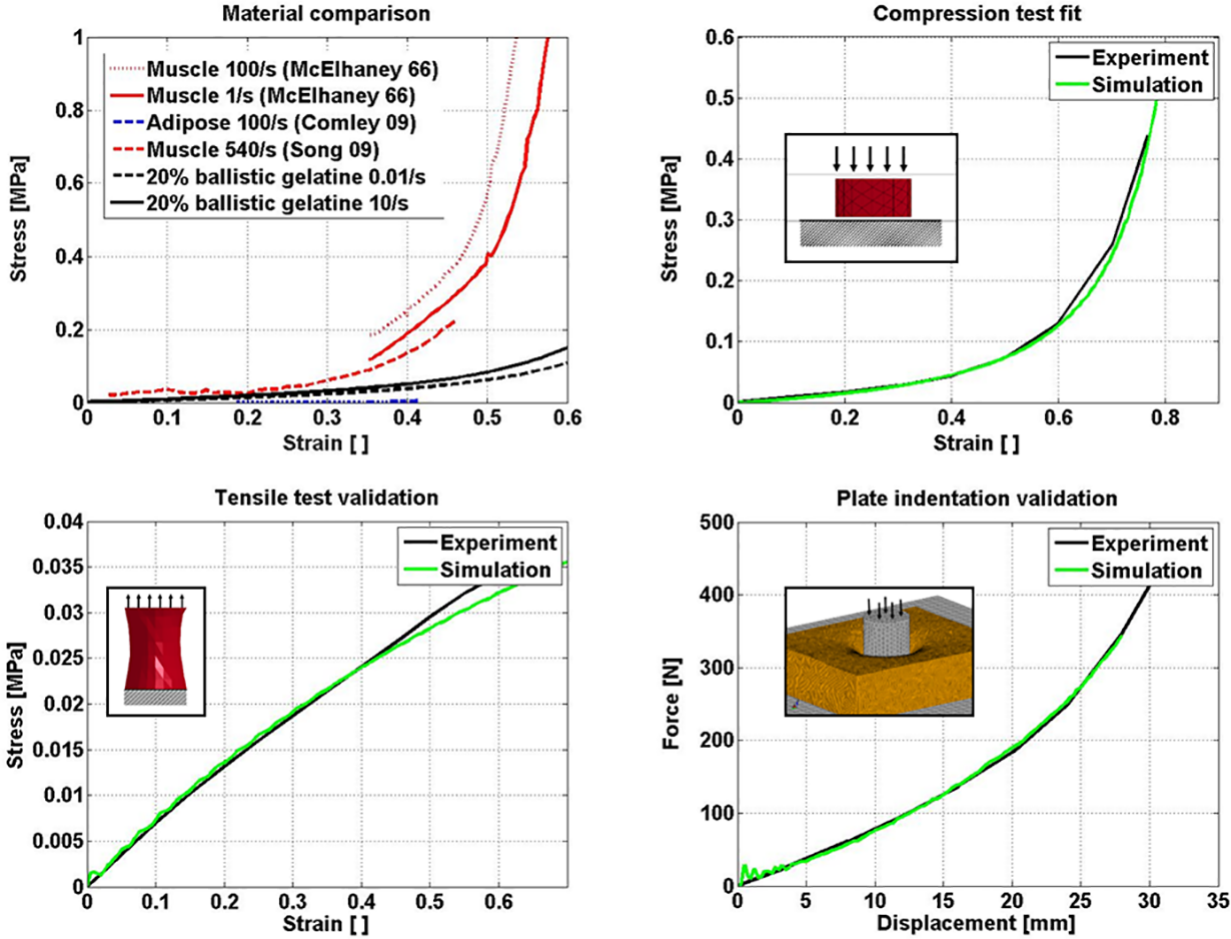
Top left: Comparison of ballistic gel, muscle [33, 34] and adipose tissue [32] response in compression. Top right: compression test results at a strain rate of 10/s compared to simulation. Bottom left: tensile test results at a strain rate of 10/s compared to simulation. Bottom right: plate indentation test results for indentation speed of 50 mm/s compared to simulation. The plate thickness was 51.6 mm.

The mixture for the ballistic gel that was used as surrogate for soft tissue was tailored so that its mechanical response would fall within the range reported in literature for muscle and adipose tissue tested in compression [32–34]. The ballistic gel mixture was tested in compression, tension, and in plate indentation, and hyper-elastic material constants reverse engineered with FEM simulation to match the outcome of the compression testing (Fig 3). Tensile tests and plate indentation were used to validate the material input under different loading conditions. The resulting Frazer-Nash rubber material model had three strain invariant coefficients (LS Dyna, MAT_031) i.e. C100 = 1.08E-2 MPa, C200 = 2.24E-4 MPa and C300 = 2.18E-6 MPa. Its density was found to be rho = 1.05 g/cm^3^ and its Poisson’s ratio was v = 0.495. Viscoelastic effects in this material were neglected.

All profiles at the lower limb constructions and the pelvic roller were assigned linear elastic material properties representative of aluminium (LS Dyna, MAT_001, E = 70 GPa, v = 0.3, rho = 2.7 g/cm^3^). Screws and guidance elements were modelled as rigid bodies. The PMMA material, in which the distal end of the femurs were potted, was modelled with a modulus of elasticity of 3 GPa and a Poisson’s ratio of 0.3 (MAT_027, σ_y_ = 20 MPa, E_tan_ = 30 MPa). The glass fibre reinforce polymer sleeves were modelled with a modulus of elasticity of 21 GPa and a Poisons’s ratio of 0.3 (LS Dyna, MAT_001). A sample FEM is shown in Fig 2.

#### Specimen alignment

The alignment of experiment and FEM was matched based on digitizer data recorded prior to the impact tests. The target anteversion and flexion angles were 13° and 37° for both legs, respectively based on literature data [22, 35, 36]. The impacted femur was fixed in 15° internal rotation, and 11° adduction to simulate an unprotected fall with the knee touching the ground after the greater trochanter had made contact with the force plate. Knee flexion was based on the calf angles reported for volunteer falls [36, 37] and lower limb segment dimensions [22]. The contralateral leg was aligned at 0° internal rotation and 0° adduction. Target values for pelvic tilt and pelvic rotation in the coronal plane were 12° [38] and 15°, respectively. This puts the sacrum in a position that simulates an upper body that is flexed away from the impact surface just prior to impact in a sideways fall [36, 39].

#### Initial conditions and boundary conditions

The global reference FEM coordinate system is shown in Fig 2. Its origin was defined at the front left corner of the force plate with the X-axis pointing upwards, normal to the impact surface. The Y-axis was defined based on the force plate edge pointing horizontally from right to left. The XY plane was the plane of fall motion. As a result, the Z-axis was an outward pointing normal to the plane of fall motion. An initial rotational velocity of 4.15 rad/s was derived based on the Optotrack data and applied as initial condition in both FEMs. This resulted in a greater trochanter velocity at impact of V_GT_ = 3.1 m/s. The translational degrees of freedom at the foot point of the impacted leg were fixed to represent the ball and socket joint constraint in the experimental setup. Gravity (9.81 m/s^2^) was applied in global X-direction. Friction was neglected for all contact surfaces, including soft tissue to force plate, femoral heads to acetabular cartilage, foot point, and metal-to-metal contact of legs. This assumption was supported by effort made during testing to minimize friction by lubrication of the force plate and ball and socket joint.

### Post processing of data

#### Accuracy of FEMs

Two types of simulations were carried out for both FEMs: first, assuming full non-linear material response of bone tissue as already described, hereafter referred to as ***non-linear*** model results; second, assuming linear response of the bone tissue i.e. excluding non-linear stress-strain response but maintaining strain rate dependency of the modulus of elasticity of the bone tissue. The outcome of these simulations will hereafter be referred to as ***linear*** model results. The purpose of this was to allow for assessment of how far into the non-linear bone material response the specimens have progressed according to the simulation results, which is an aspect of hip biomechanics that cannot easily be studied with experiments alone.

For both non-linear and linear models the following comparison between experimental and corresponding computational results was carried out:

- Impact force over time response at the level of the force plate and error in predicted peak impact force.
- Pelvic ring deformation (**C**_**LR**_ **= LR1—LR2**) derived from the HS video data was compared to displacements in the FEMs at the LR1 and LR2 marker locations and errors in predicted **C**_**LR**_ at the time of experimentally measured peak impact force quantified. The location of these markers are comparable, albeit not identical, to the lateral ring markers in the experimental study by Beasson et al. [21].
- Pelvic rotation in the coronal plane and errors in predicted pelvic rotation at the time of experimentally measured peak impact force quantified.
- Impact load over displacement (**k**_**ST1,lo**_) of right GT soft tissue marker (ST1) was evaluated at 300–1000 N, which corresponds to effective pelvic stiffness reported in the literature [10].

### Internal reaction forces and energy absorption in the hip

Force transducers were embedded in the FEMs in the middle of the femoral neck, at the interfaces between femoral head and acetabular cartilage of both hip joints, the pubic symphysis and the sacrum. The internal reaction forces were assessed for both linear and non-linear models.

To monitor the energy absorption in the models, strain energy stored in the soft tissue, femur on the impact side, and pelvis was quantified at all simulation time points for both linear and non-linear models. The energy absorbed by the pelvis includes the energy absorbed by the bones, cartilage and ligaments. The remaining kinetic energy was also quantified to allow for monitoring of the energy balance after impact.

### FEM based fracture prediction

The predicted strain pattern in the femur on the impact side 2 ms after time of computational measured peak force was used to predict fracture outcomes. At this time point, unloading or failure of the femur is visible in its element strains. Failure was assumed to be possible when the 1^st^ and 3rd principal strain component of the St.Vernant-Green strain tensor was higher than 1.4% and lower than -2% for tension and compression, respectively. [40] These values correspond to the onset of element softening according to the material mapping strategy.

## Results

### Accuracy of FEMs

**Fig 4:**
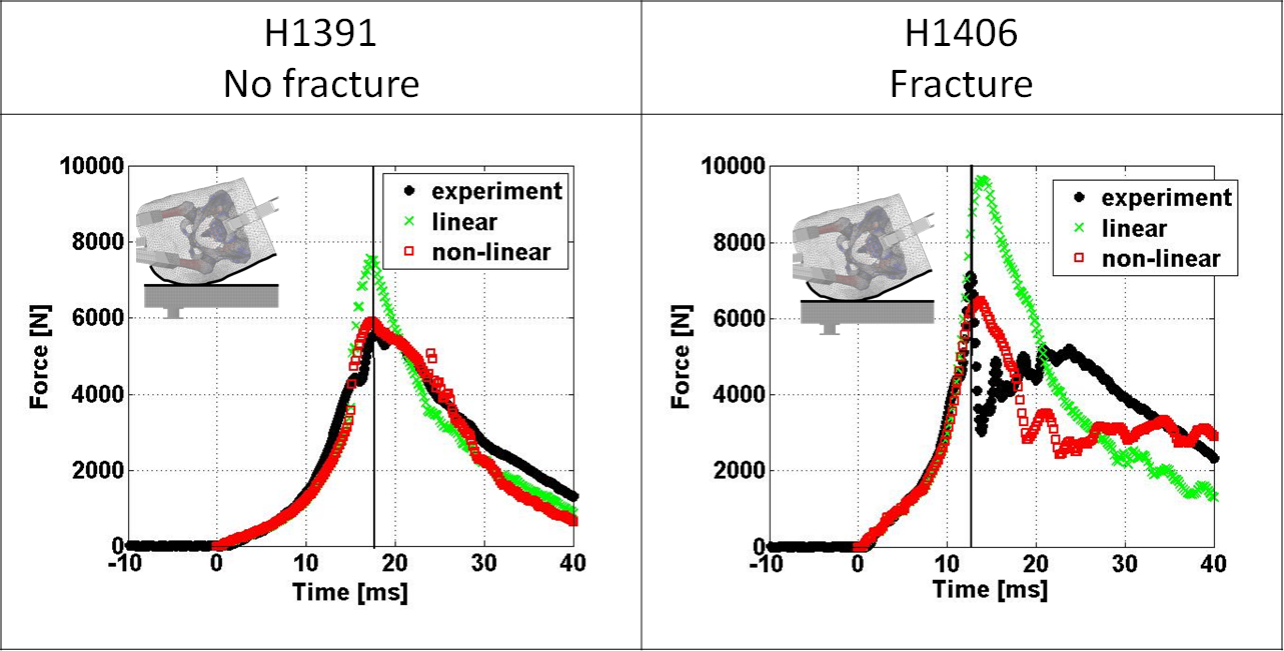
Comparison of impact force over time response between experimental FEM derived results. The vertical lines indicate the time of experimentally measured peak impact force.

The comparison of impact force over time between experimentally derived and FEM derived results is illustrated in Fig 4 for both linear and non-linear FEMs. Both model types were able to closely match the experimental response until approaching the peak impact force where the non-linear models are in good agreement with the experimental results but linear models over predict the response. Beyond the peak impact force both linear and non-linear models are able to conceptually match the experimental response for the non-fracture specimen (H1391). For the fractured specimens (H1406) the FEMs are not able to capture the fast drop in force that occurred during the experimental testing. The errors in predicted peak impact forces are provided in Table 2.

**Table 2:** Comparison of experimentally and FEM derived results.

	Experiment	linear FEM	Non-linear FEM	Error linear	Error non-linear
Peak impact force H1391	5641 N	7573 N	5903 N	34.2%	4.6%
Peak impact force H1406	7132 N	9655 N	6454 N	35.4%	-9.5%
Pelvic ring deformation (**C**_**LR**_) at peak impact force H1391	2.5 mm	2.3 mm	3.4 mm	-8.0%	36.0%
Pelvic ring deformation (**C**_**LR**_) at peak impact force H1406	1.1 mm	1.6 mm	1.1 mm	45.5%	0.0%
Pelvic rotation at peak impact force H1391	6.8	6.6	6.2	-2.9%	-8.8%
Pelvic rotation at peak impact force H1406	3.8	4.5	4.1	18.4%	7.9%
Effective pelvic stiffness in the range of 300-1000N (**K**_**ST1,lo**_) for H1391	50.7 N/mm	47.4 N/mm	46.9 N/mm	-6.5%	-12.2%
Effective pelvic stiffness in the range of 300-1000N (**K**_**ST1,lo**_) for H1406	80.3 N/mm	77.3 N/mm	79.8 N/mm	-3.8%	-0.7%

**Fig 5:**
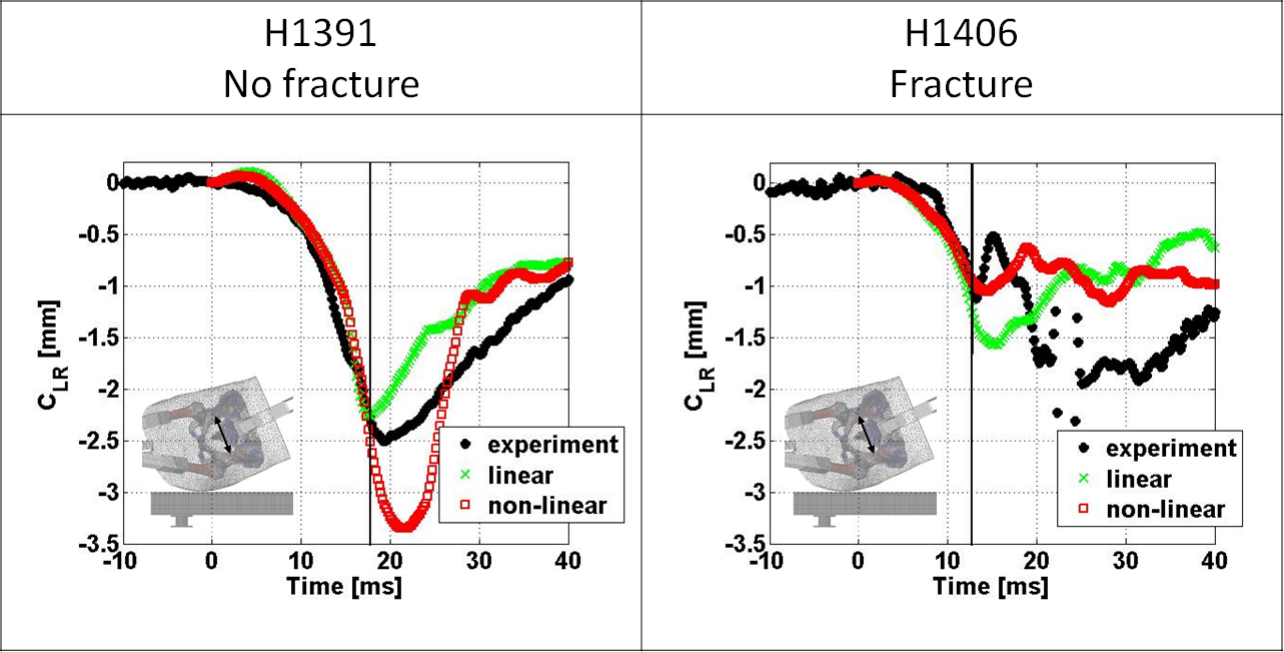
Comparison of pelvic ring deformation over time between experimentally and both linear and non-linear FEM derived results. The vertical lines indicate the time of experimentally measured peak impact force.

The comparison of pelvic brim compression between experimentally derived and FEM derived results is illustrated in Fig 5 for both, linear and non-linear FEMs. Both model types were able to closely match the experimental response for both specimens until approaching the time of peak impact force. Beyond the time of peak impact force the non-linear models overestimate the response but the linear models underestimate the response for specimen H1391. Both models deviate considerably in their prediction of pelvic brim compression after peak impact force and fracture of specimen H1406. The errors in predicted lateral ring compression at the time of peak impact force are provided in Table 2.

**Fig 6:**
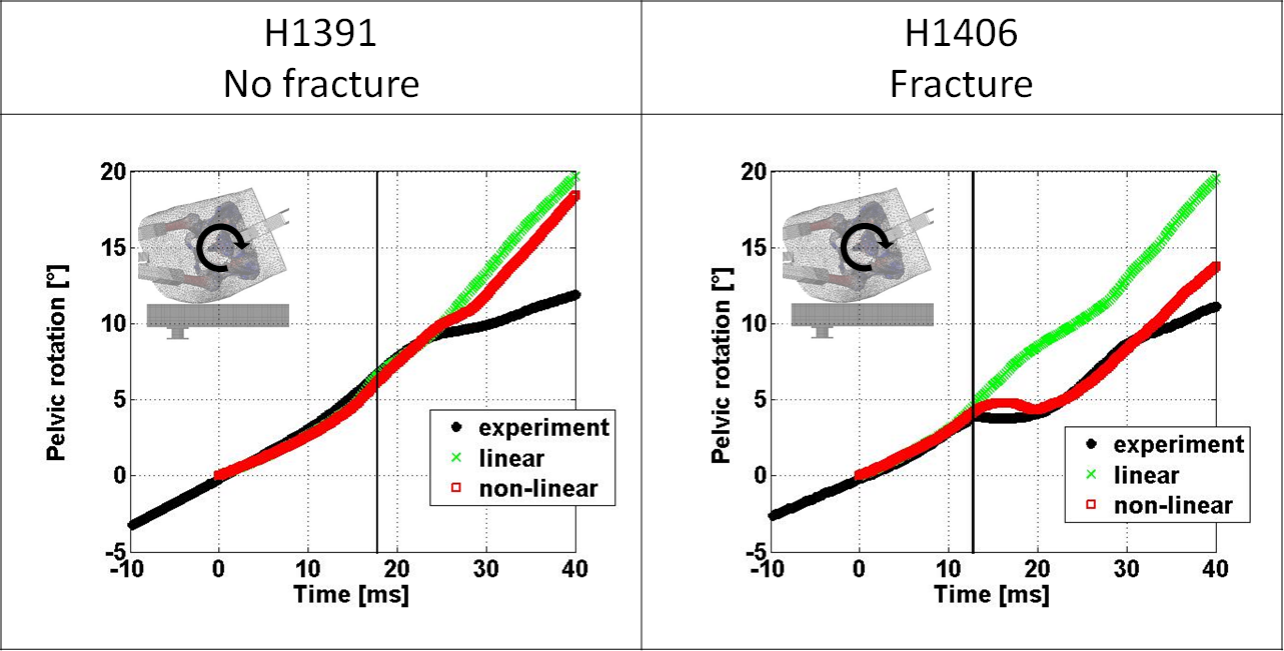
Comparison of pelvic rotation in the coronal plane over time between experimentally and FEM derived results. The vertical lines indicate the time of experimentally measured peak impact force.

The comparison of pelvic rotation in the coronal plane between experimentally derived and FEM derived results is illustrated in Fig 6 for both linear and non-linear FEMs. Both model types were able to closely match the experimental response to the time of peak impact force for both specimens and in fact as far as 5 ms beyond the time of peak impact force for specimen H1391. Beyond the time of peak impact force the linear model over-estimates the pelvic rotation for specimen H1406 but the non-linear models capture the response at least qualitatively. The errors in predicted pelvic rotation at the time of peak impact force are provided in Table 2.

**Fig 7:**
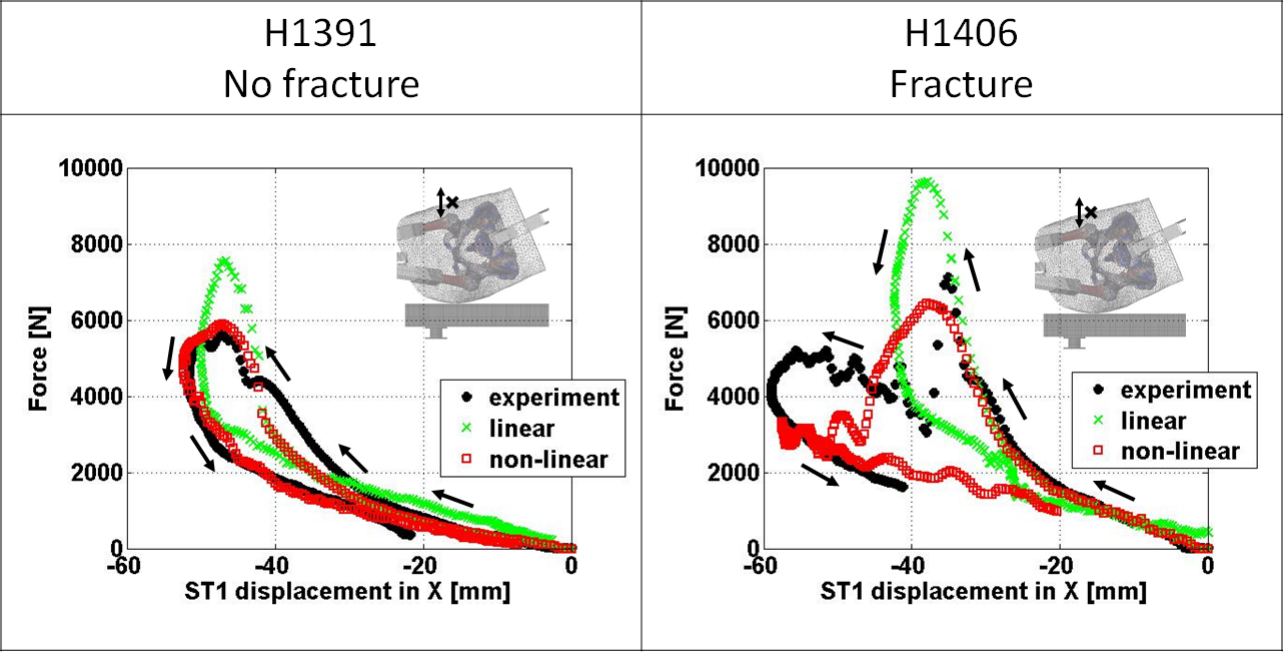
Comparison of impact force over displacement in x-direction of contralateral GT soft tissue marker (ST1) in the vertical direction between experimentally and FEM derived results.

The comparison of impact load plotted against the displacement of contralateral GT soft tissue marker (ST1) based on experimentally derived and FEM derived results is illustrated in Fig 7 for both linear and non-linear FEMs. Both model types were able to closely match the experimental response until approaching peak impact force for both specimens The predicted effective pelvic stiffness at low force range (**k**_**ST1,lo**_) is provided in Table 2.

### Internal reaction forces and energy absorption in the hip

**Fig 8:**
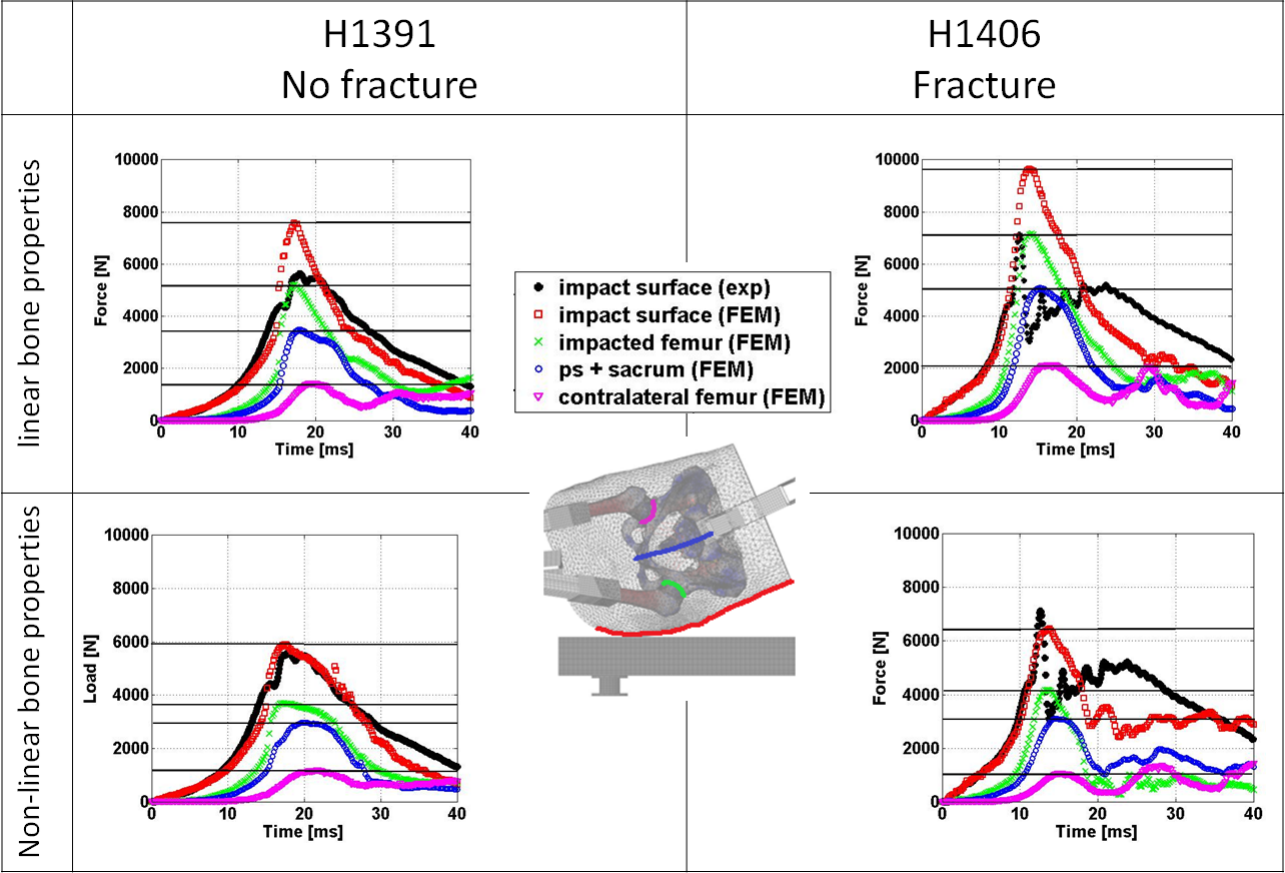
Comparison of internal reaction forces in the hip over time between experimentally and FEM derived results. Linear FEM results are in the top row and non-linear FEM results in the bottom row. Horizontal lines mark the peak forces recorded for the different evaluation sites in the FEM.

The internal reaction forces in the hip during impact, derived from the linear and non-linear FEM results, are illustrated in Fig 8. The loads transferred through the acetabular contact interfaces were found to differ by less than 1% from the force transferred through the corresponding femoral necks. Thus, only contact forces at the hip joints are reported. Decreasing peak impact forces as well as a time lag in peak impact forces with increasing distance from the impact site could be observed for both specimens and in both types of FEMs. For specimen H1391, the peak impact force in the femur on the impact side was found to be 68.7% and 62.4% of the peak impact force between soft tissue and force plate in the linear and non-linear models respectively. The corresponding numbers for H1406 were 74.3% and 65.2%. The internal reaction forces in the pelvis for both specimens and both models indicated almost equal load sharing between the pubic symphysis joint and the sacrum. The peak impact forces transferred through the contralateral femurs according to the linear simulation results were 18.7% and 22.1% of the peak impact forces at the force plate for specimens H1391 and H1406, respectively. Corresponding numbers were 20.0 and 16.6% for the non-linear models.

**Fig 9:**
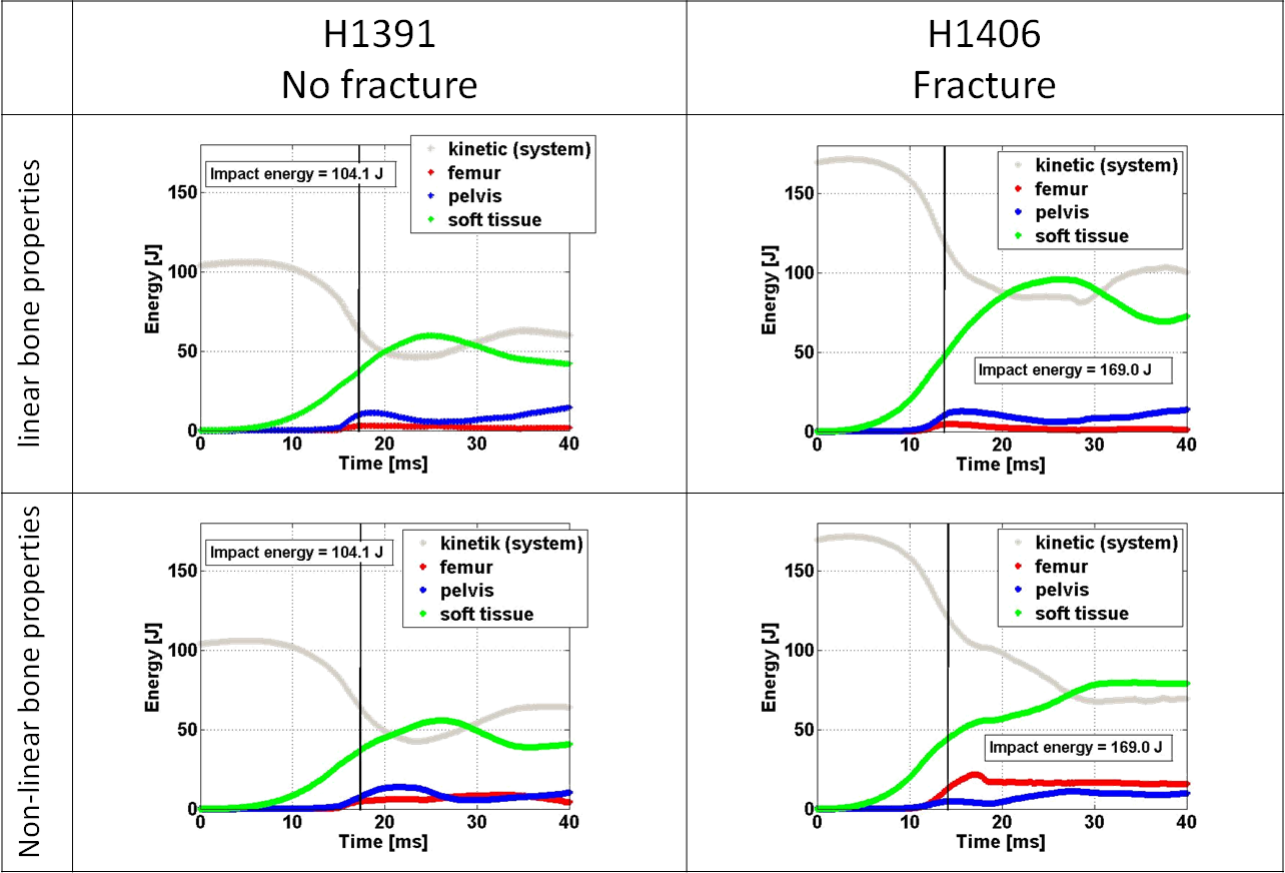
**Energy absorption of the main structures of the specimens during impact according to simulation results for linear FEMs (top row) and non-linear FEMs (bottom row)**. The vertical lines indicate the time of computationally measured peak impact force.

Energy absorption in the FEMs is illustrated in Fig 9. Based on the linear FEM results, for both specimens, the soft tissue absorbed the largest portion of the energy at the time of experimental peak impact force. The total energy in the system for specimen H1391 at peak force was 115.3 J, corresponding to 104.1 J of kinetic energy released at the time of impact and 11.2 J of additional potential energy released until the time of peak impact force. For the linear FEMs the distribution of the 115.3 J at the time of peak force was 2.4%, 8.7%, 32.3% and 54.5% in the femur, pelvis, soft tissue and as kinetic energy, respectively. The corresponding distribution of the 183.6 J of total energy for specimen H1406 at the time of peak force was 2.4%, 5.7%, 25.9% and 64.7%. For the non-linear FEMs the distribution of the 115.3 J was 4.2%, 6.7%, 32.2% and 54.9% in the femur, pelvis, soft tissue and as kinetic energy, respectively. The corresponding distribution of the 183.6 J of total energy for the non-linear FEMs of specimen H1406 was 7.0%, 2.6%, 24.2% and 65.3%.

### FEM based fracture prediction

**Fig 10:**
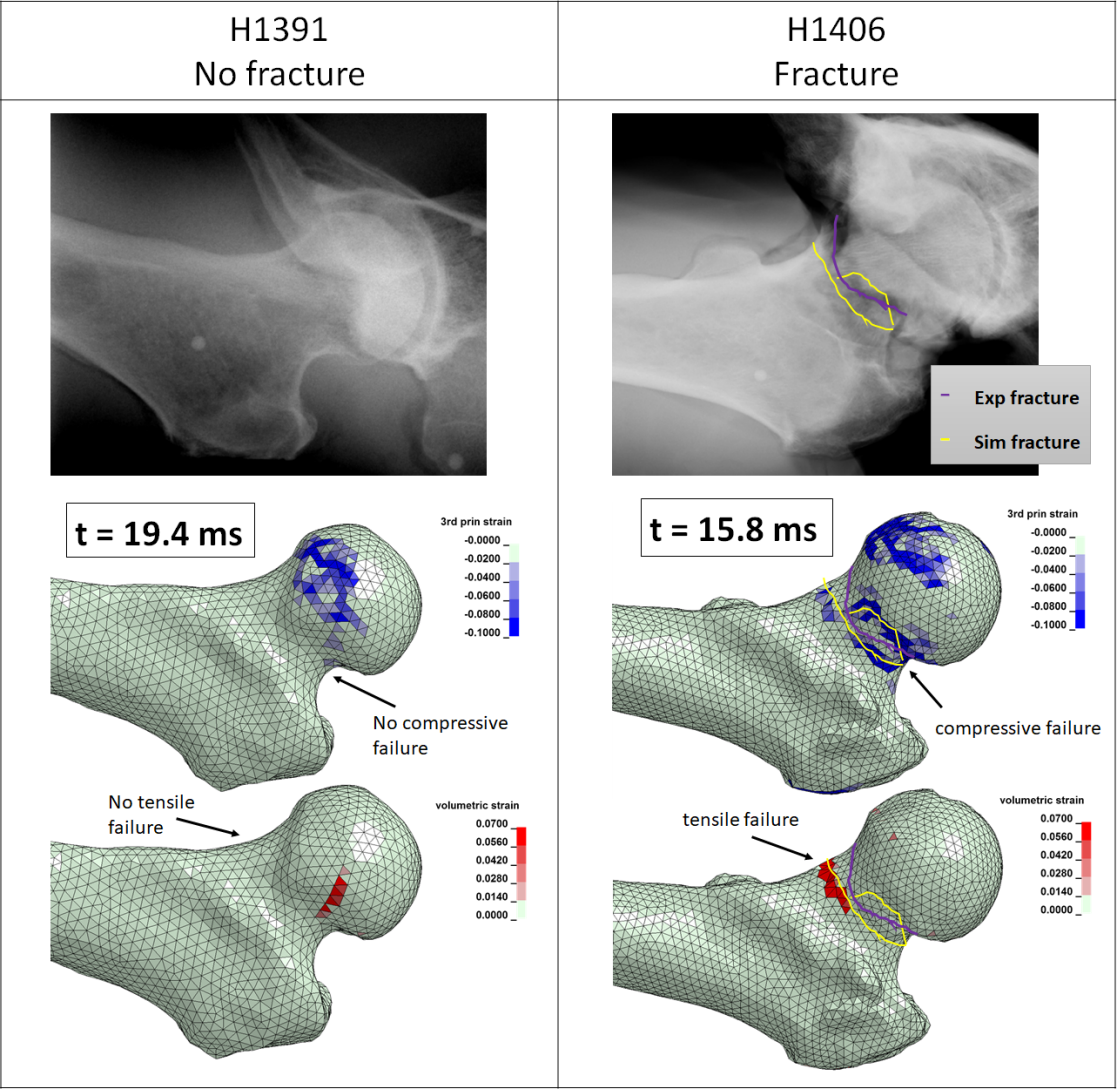
FEM based fracture prediction based on surface strain patterns as per simulation results 2 ms after the time of experimentally measured peak impact force. Experimental fracture line marked in purple and FE predicted fracture line marked in yellow. Top row: Post-testing X-rays. Middle row: FEM derived 3rd principal strain component of the St.Vernant-Green strain tensor. Bottom row: FEM derived 1st principal strain component of the St.Vernant-Green strain tensor.

The results of the FEM based fracture prediction are illustrated in Fig 10 for non-linear FEMs only. For Specimen H1406, damage initiated inside the femur in the trabecular structure in compression then progressed to failure at the superior aspect of the femoral neck in compression and finally failure at the inferior aspect of the femoral neck in tension, indicated by positive volumetric surface strain, led to catastrophic failure of the femur. This occurred after severe strain levels being reached within the cancellous bone compartments within the femoral neck and the intertrochanteric region (see FEM derived video data supplied as supplementary material). The location of the surface crack according to the FEM results matched the fracture pattern visible in the post testing x-ray of the specimen well (Fig 10). For specimen H1391, surface strains did not indicate failure in compression or in tension. However, damage had started in the trabecular volume inside the greater trochanter and femoral neck of the femur. FEM simulations for both specimens show high compressive strains (3rd principal strains) in the area where the femoral head is pressing into the acetabular cartilage, however the post testing x-rays and inspection of the specimens in the lab could neither confirm or refute compressive damage in the femoral head.

## Discussion

The aim of the present study was to develop a methodology for constructing and assessing the accuracy of explicit FEMs for the biofielic simulation of sideways falls, to use these FEMs for quantifying internal reaction forces and energy absorption in the hip during the impact, and to assess the ability of these models to separate a femoral fracture endpoint from a non-fracture endpoint. We found the FEM-derived results to closely match the experimental test results approaching the time of peak impact force and to accurately predict femur fracture in the specimen that fractured and predict no fracture in the specimen that didn’t fracture. We found the force transferred through the femoral neck to be significantly lower than the impact force measured between soft tissue and ground for both specimens. We found the soft tissue to be the component that absorbed the largest part of the energy of the tissue types in the hip region. Finally, we found surface strain patterns derived from FEM results to match observations based on post testing x-rays of the specimens.

The simulation results show that the non-linear FEMs were able to closely match the measured impact kinetics and kinematics up to the time of peak impact force, which is important with respect to the potential use of these types of FEMs for fracture risk prediction. The FEMs with the linear material properties on the other hand over predict peak impact force by roughly 25%. Based on this outcome it is tempting to speculate that this indicates that even specimens that do not fracture during impact may be loaded considerably into the non-linear response of the bone tissue. This is indirectly supported by studies that have found bone failure in the proximal femur to initiate in the cancellous bone and not on the surface of the cortical bone [41, 42]. This would highlight the importance of ductility of bone tissue for surviving a fall to the hip but ductility of human bone was found to decrease with age [43].

The measured effective pelvic stiffness at low force range for our specimens was found to be 50.3 and 80.7 N/mm for H1391 and H1406 respectively (Table 2). It falls within the range of effective pelvic stiffness range reported in the literature i.e. 20.9–90.0 N/mm depending on protocol [10, 44, 45]. The maximum effective pelvic stiffness at high force range is approximately 5 times higher which highlights the limitation of using effective pelvic stiffness as determined per low drop height volunteer testing for predicting impact forces at injurious load levels. The FEMs were able to capture this effect which points to the potential for using such FEMs to systematically predict impact forces resulting from falls.

Our FEM derived results on energy absorption are, to our knowledge, the first quantitative evaluation of the energy balance during sideways fall impact. It is interesting to note that at the time of peak impact force only 4.4% of the total energy is absorbed by the femur on the impact side of specimen H1391, that did not fracture. At this time point, roughly 50% of the total energy is still in the form of kinetic energy. This speaks towards the importance of a given femur being able to hold the load while the energy is transferred to other tissue structures, if it is to survive an impact after a fall. It is interesting to note that the pelvis absorbs less energy than the femur in the specimen that fractured (H1406), which is opposite to what occurred in the specimen that did not fracture (H1391). Compared to studies with surrogate systems but real soft tissues, the maximum amount of energy absorbed by the soft tissue in our analysis was either comparable or higher. [15] Results for the present two models indicate that the amount of energy that was transferred to the soft tissues varied according to the subject, even for similar soft tissue thicknesses. An important difference of the surrogate system compared to real soft tissue in reference Robinovitch et al. (15), is that all kinetic energy had to be taken up by their system at peak force, while in our system, large amounts of the energy in the fall was still kinetic, as linear and rotational velocity, at the time of peak force. Testing and simulations of more specimens will reveal whether this is a general trend or not.

The fracture prediction based on the FEM results indicates that this type of modelling holds considerable promise with respect to being able to separate a fracture endpoint from a non-fracture endpoint. The patterns of severe strains according to simulation match fracture location in specimen H1406 relatively well while strain patterns in H1391 give no clear indication of a failure which is in line with the outcome of the experimental testing. This is in agreement with the findings of our previous studies on dynamic FEMs of the proximal femur i.e. that the predicted regions of severe strains match experimentally induced fracture patterns relatively well [8, 46]. Further, our validated femur model predicted the fracture and non-fracture that was experimentally observed while the FE was closely matching the impact load measured at the force plate.

Since we cannot measure the internal reaction forces through pelvic region experimentally without compromising tissues structures, we cannot directly verify our findings related to force attenuation between the level of the force plate and the forces transferred through the acetabular cups. However, our FEM results are in agreement with previous literature that reports the magnitude of the force that is transferred through the impacted femur to be significantly lower than the impact force measured at the level of the force plate [13].

There are several limitations associated with our study that need to be addressed. First, we have tested and modelled only two specimens, which obviously limits our ability to draw strong general conclusion on what predisposes a hip to fracture or how a femur that doesn’t fracture is different than one that does. Second, we are simulating an experimental setup that is using a surrogate soft tissue. While the upside of that setup is a full control over the properties of that important part of these models, the downside is that the surrogate tissue may not accurately reflect the heterogeneity of the soft tissues in the human body. Third, we are simulating a fall to the ground assuming passive muscles but low drop height studies indicate that muscle activation may influence the magnitude of the impact force. The force measured at femoral neck may also be influenced by muscle activation. Using a surrogate human model Choi et al. [47] found muscle activation to influence the stresses in the femoral neck. The strength of our study is that a close agreement of the FEM with the cadaveric experiment up to the point of peak impact force could be achieved using only input based on experiments, CT data and literature. No parameters were optimized to match the FEM to the results of the experimental fall simulation.

In summary, we developed a novel modelling technique for in-silico simulation of a fall to the hip from standing. The model results were found to accurately represent experimental data in terms of impact force to peak, displacements and fracture pattern to time of peak impact. Using this modelling technique, we were able to assess the internal reaction forces through the hip during impact which is currently not possible in ex vivo tests. This is the first study with quantitative data on the energy absorption in the pelvic region during sideways falls.

## Supporting information

S1 Detailed specimen information Detailed information on specimen geometry, impact energy, masses of system components, and partial mass moments of inertia. (DOCX)
