# Correction: On the internal reaction forces, energy absorption, and fracture in the hip during simulated sideways fall impact

**DOI:** 10.1371/journal.pone.0208286

**Published:** 2018-11-26

**Authors:** Ingmar Fleps, William S. Enns-Bray, Pierre Guy, Stephen J. Ferguson, Peter A. Cripton, Benedikt Helgason

In Fig 2, the markers PB1 and PB2 are incorrect. PB1 should be labeled LR1 and PB2 should be labeled LR2. Additionally, the marker d_PB_ should be removed. Please see the corrected Fig 2 here.

**Fig 2:**
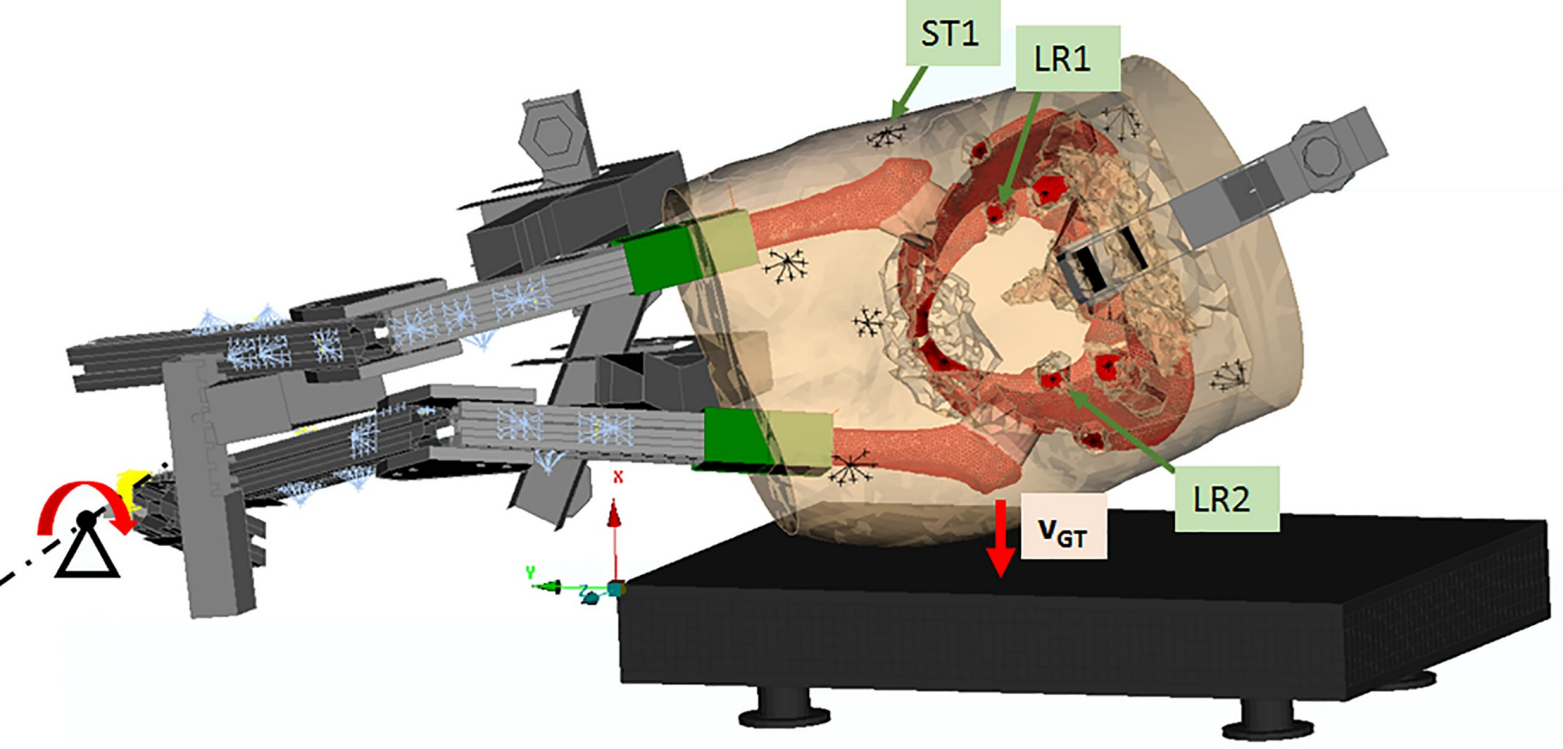
FEM of specimen H1391 aligned in initial position corresponding to the time of first contact with force plate during the experimental testing. Lower limb constructions coloured grey, lower limb masses coloured blue and soft tissue coloured orange. Marker points corresponding to location of video markers of the physical specimen are shown in black. Marker ST1 corresponds to a marker attached to the surrogate soft tissue above the right GT. Markers LR1 and LR2 correspond to the position of markers attached to the right and left side of the pelvic ring, respectively.
