# Supplementary material for: On the internal reaction forces, energy absorption, and fracture in the hip during simulated sideways fall impact
Source: PLoS One. 2018 Aug 16;13(8):e0200952. doi: 10.1371/journal.pone.0200952 (PMC6095517; doi:10.1371/journal.pone.0200952)
Supplement: S1 Detailed specimen information — (DOCX) [file pone.0200952.s001.docx]

| Parameters | Unit | H1391 | H1406 |
| --- | --- | --- | --- |
| Gender |  | Female | Male |
| Age | [years] | 62 | 94 |
| Height | [m] | 1.65 | 1.73 |
| Body mass | [kg] | 59.0 | 83.9 |
| Femoral neck aBMD | [g/cm^2^] | 0.694 | 0.697 |
| T-score | [ ] | -0.91 | -0.88 |
| Width of pelvic inlet | [mm] | 125 | 134 |
| Distance between femoral head centres | [mm] | 162 | 188 |
| Distance between greater trochanters* | [mm] | 265 | 323 |
| Hip width** | [mm] | 332 | 384 |
| Greater trochanter soft tissue thickness | [mm] | 32 | 31 |
| Rotational impact velocity | [1/s] | 4.13 | 4.15 |
| Inertia I_Z_ with respect to foot point | [kg*m^2^] | 12.9 | 19.6 |
| Specimen mass, pendulum | [kg] | 30.56 | 44.35 |
| Rotational energy, pendulum | [J] | 104.1 | 169.0 |
| Lower limb construction mass (impacted leg) | [kg] | 6.09 | 8.40 |
| Lower limb construction mass (contralateral leg) | [kg] | 6.00 | 8.51 |
| Mass pelvic roller | [kg] | 0.77 | 0.77 |
| Mass cadaveric specimen*** | [kg] | 1.78 | 2.49 |
| Surrogate soft tissue mass | [kg] | 15.34 | 23.70 |
| I_Z_ lower limb construction (impacted leg)**** | [Kg/m^2^] | 0.90 | 1.26 |
| I_Z_ lower limb construction (contralateral leg)**** | [Kg/m^2^] | 0.94 | 1.30 |
| I_Z_ pelvic roller**** | [Kg/m^2^] | 0.79 | 0.80 |
| I_Z_ cadaveric specimen*** ^,^ **** | [Kg/m^2^] | 1.02 | 1.47 |
| I_Z_ surrogate soft tissue**** | [Kg/m^2^] | 9.24 | 14.87 |

* In fall alignment

** widest distance, not the distance at the greater trochanters

*** bones, cartilage and ligament with femoral potting and square tubes

**** inertia with respect axis of rotation of the pendulum
